# The population and landscape genetics of the European badger (*Meles meles*) in Ireland

**DOI:** 10.1002/ece3.4498

**Published:** 2018-09-12

**Authors:** Jimena Guerrero, Andrew W. Byrne, John Lavery, Eleanor Presho, Gavin Kelly, Emily A. Courcier, James O'Keeffe, Ursula Fogarty, Denise B. O'Meara, Dennis Ensing, Carl McCormick, Roman Biek, Robin A. Skuce, Adrian R. Allen

**Affiliations:** ^1^ Centre D'Ecologie Fonctionelle et Evolutive CEFE‐CNRS Montpellier France; ^2^ Veterinary Sciences Division Agri‐Food and Biosciences Institute (AFBI) Belfast UK; ^3^ Department of Agriculture, Environment and Rural Affairs Northern Ireland (DAERA‐NI) Veterinary Epidemiology Unit Belfast UK; ^4^ Department of Agriculture Food and the Marine (DAFM) Dublin Ireland; ^5^ Irish Equine Centre Johnstown Ireland; ^6^ Department of Chemical and Life Sciences Waterford Institute of Technology Waterford Ireland; ^7^ Agriculture, Sustainable Agri‐Food Sciences Division Agri‐Food and Biosciences Institute Belfast UK; ^8^ Institute of Biodiversity Animal Health and Comparative Medicine University of Glasgow Glasgow UK

**Keywords:** dispersal, gene flow, landscape, population structure

## Abstract

The population genetic structure of free‐ranging species is expected to reflect landscape‐level effects. Quantifying the role of these factors and their relative contribution often has important implications for wildlife management. The population genetics of the European badger (*Meles meles*) have received considerable attention, not least because the species acts as a potential wildlife reservoir for bovine tuberculosis (bTB) in Britain and Ireland. Herein, we detail the most comprehensive population and landscape genetic study of the badger in Ireland to date—comprised of 454 Irish badger samples, genotyped at 14 microsatellite loci. Bayesian and multivariate clustering methods demonstrated continuous clinal variation across the island, with potentially distinct differentiation observed in Northern Ireland. Landscape genetic analyses identified geographic distance and elevation as the primary drivers of genetic differentiation, in keeping with badgers exhibiting high levels of philopatry. Other factors hypothesized to affect gene flow, including earth worm habitat suitability, land cover type, and the River Shannon, had little to no detectable effect. By providing a more accurate picture of badger population structure and the factors effecting it, these data can guide current efforts to manage the species in Ireland and to better understand its role in bTB.

## 1 INTRODUCTION

A major force likely to have affected population genetic structure of species is the effects of abiotic and biotic landscape features on gene flow. As an emerging discipline, landscape genetics combines population genetics and landscape ecology to assess the influence of landscape or environmental features on genetic variation in wildlife populations, their dispersal, and connectivity of habitats (Manel & Holderegger, 2013). This can provide key information for wildlife management, including cases where species act as reservoirs or vectors of pathogen infection (Frantz, Pope, Etherington, Wilson, & Burke, 2010; Kierepka & Latch, 2016a,b; Pope, Domingo‐Roura, Erven, & Burke, 2006).

**Figure 1:**
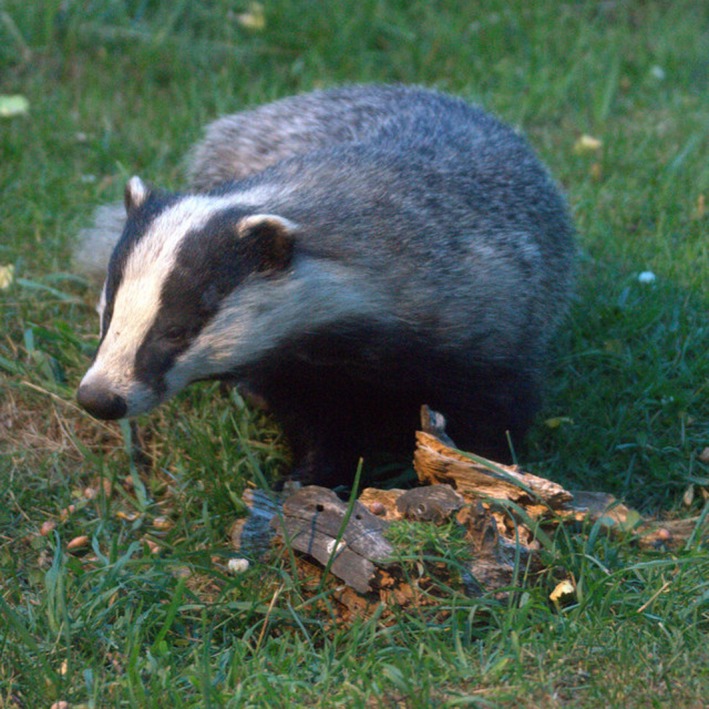
European badger (*Meles meles*) Photo © Mike Pennington (cc‐by‐sa/2.0)

The European badger (*Meles meles*) Figure 1 is the largest terrestrial carnivore in Britain and Ireland, and is of significant ecological (e.g., ecosystem engineer) and economic importance (as a suspected reservoir of bovine tuberculosis, bTB) across this territory (Roper, 2010). A significant body of work has been undertaken to elucidate the species’ role in cattle bTB epidemiology (Roper, 2010). The species’ genetic structure at continental scale has been studied extensively (Del Cerro, Fernando, Chaschin, Taberlet, & Bosch, 2010; Frantz et al., 2014; Marmi et al., 2006; O'Meara et al., 2012). In contrast, there is limited information available on badger population genetics at the national scale, which is likely to be the scale most relevant to management. To date, there has been no large island‐wide survey of the genetic structure of the badger in Ireland.

Previously, studies from across Europe have noted that badgers exhibit limited dispersal/philopatry (Pope et al., 2006). Dispersal distance seems to be inversely proportional to population density, with a large proportion of individuals exhibiting philopatry at high densities (Frantz, Cellina, Krier, Schley, & Burke, 2009). Although badger densities in Ireland are typically not as high as those in southern Britain (e.g., Woodchester Park; 0.4 setts/km^2^ vs. 2.88 setts/km^2^, respectively), they can still be considered relatively high compared to other European populations (Byrne, Sleeman, O'Keefe, & Davenport, 2012; Pope et al., 2006). It is also noteworthy that in Ireland, whilst general philopatry appears to hold, mark–recapture studies of Irish badgers have documented rare long‐distance dispersal of up to 22.1 km (Byrne, Quinn, et al., 2014).

Aside from geographic distance, other landscape features likely affect gene flow of badgers. Water bodies and motorways have been observed to hinder European badger gene flow (Frantz et al., 2010). Furthermore, badgers have generally been recorded at low altitudes (<200 m; Byrne et al., 2012), and their abundance, habitat selection, and foraging behavior are positively associated with land use categories such as pasture, forested areas, and grasslands—urban and arable land are generally avoided (Byrne et al., 2012; Hammond, McGrath, & Martin, 2001).

On the other hand, the effect of biotic interactions on the gene flow of organisms in general has been little studied (Hand, Lowe, Kovach, Muhlfeld, & Luikart, 2015) regardless of the crucial insights that such research could provide. In this sense, badgers are a particularly interesting system because they are generally assumed to be earthworm (*Lumbricus terrestris*) specialists (Kruuk & Parish, 1981; Muldowney, Curry, O'Keefe, & Schmidt, 2003), which could result in gene flow being strongly affected by earthworm availability. Conversely, there is some indication that in Ireland, the diet of the badger varies seasonally and is less reliant on earthworms than observed elsewhere (Cleary, Corner, O'Keefe, & Marples, 2009) which could result in little effect of prey availability on gene flow.

In light of the above, applying landscape genetics to study the effect of landscape features and biotic interactions on badger gene flow may help to inform more fully on the ecology of the species in Ireland. The latter could be of benefit in developing a better understanding of how/whether badger population structure influences bovine tuberculosis epidemiology. In this study, therefore, we aim to provide the first comprehensive, large scale assessment of genetic population structure of the badger across Ireland and to identify the landscape features which have likely shaped it. Specifically, we studied the influence of geographic distance, landscape variables (elevation, land cover, Ireland's only continental scale river: the Shannon), and biotic interactions (earthworm availability), on badger gene flow.

## 2 MATERIALS AND METHODS

### 2.1 Sample collection

A total of 454 badger samples were collected from the Republic of Ireland (RoI) and Northern Ireland (NI; Supporting Information Table S1). Badger carcasses (*n* = 176) from an ongoing road traffic accident (RTA) survey were collected by the Department of Agriculture, Environment and Rural Affairs Northern Ireland (DAERA‐NI) across all six counties of NI during the period from September 2011 to March 2013. GPS locations of all carcasses were logged and a tissue sample was stored for DNA extraction. In addition, badger carcasses (*n* = 278) from ongoing culling efforts in the RoI were collected by the Department of Agriculture, Food and the Marine (DAFM) during 2014. These badger carcasses were collected from sites distributed across 23 of the 26 counties of RoI. The three counties excluded were Donegal, Dublin, and Louth on account of there being no badger carcases available during the time window described. GPS coordinates of locations of culled animals were collected and a tissue sample stored for DNA extraction. For 21 samples across both territories, there were no geo‐location data available. Samples were frozen at −20°C before DNA extraction.

**Figure 2:**
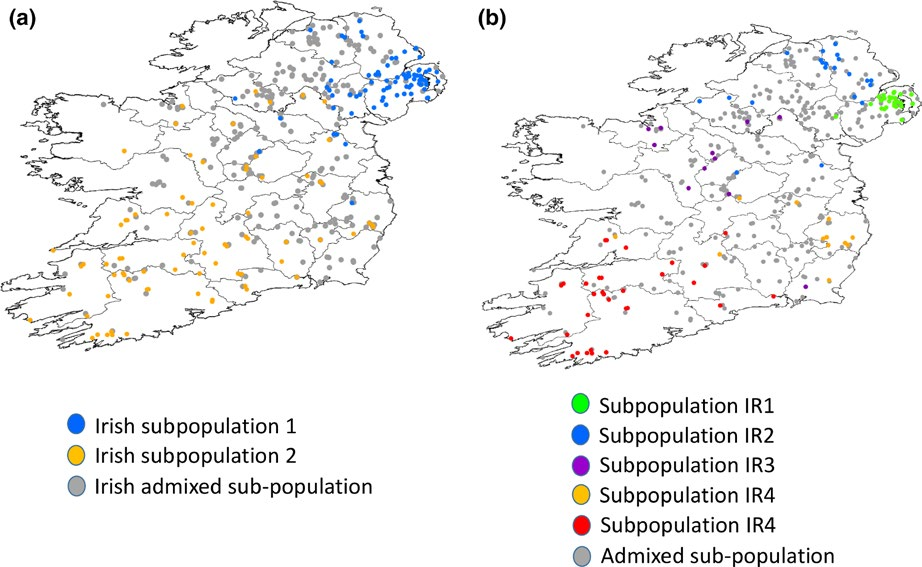
(a) STRUCTURE microsatellite analysis *K* = 2 spatial distribution for all 454 Irish badgers. (b) STRUCTURE microsatellite analysis *K* = 5 spatial distribution for all Irish badgers

For a full breakdown of numbers of animals submitted per county across Ireland, see Supporting Information Table S1. GPS locations for all animals are found in Supporting Information Data S1. The map in Figure 2a illustrates the position of all badgers sampled. A map detailing the geographic position and names of all Irish counties and the position of the River Shannon is shown in Supporting Information Figure S1.

### 2.2 DNA extraction

We extracted DNA from all tissue samples using a Qiagen DNeasy tissue mini kit (Qiagen, Crawley, West Sussex, United Kingdom). Extracted DNA was stored at −20°C until PCR amplification for either microsatellite or mitochondrial DNA sequence analysis.

### 2.3 Microsatellite genotyping

We genotyped all 454 samples at the following 14 microsatellite loci—Mel102, Mel103, Mel104, Mel105, Mel106, Mel109, Mel110, Mel111, Mel112, Mel113, Mel114, Mel115, Mel117, and Mel129 (Carpenter et al., 2003). Forward primers were 5′ end‐labeled with a fluorescent tag—6FAM (blue) for Mel104, Mel105, Mel106, Mel109, Mel117, and Mel129; Hex (green) for Mel102, Mel103, Mel110, Mel113, and Mel115; Cy3 (yellow) for Mel111, Mel112, and Mel114. All primers were supplied by Sigma‐Aldrich (Dorset, United Kingdom).

The 14 primer pairs were arranged into three multiplex PCR. X10 multi primer master mixes were constituted as follows: Mix 1 Mel106 and Mel104—2 μM unlabeled primers, 1 μM labeled primers; Mel111 and Mel112—8 μM unlabeled primers, 4 μM labeled primers; Mel109 and Mel117—1 μM unlabeled primers, 0.5 μM labeled primers. Mix 2 Mel105 and Mel129—4 μM unlabeled primers, 2 μM labeled primers; Mel102 and Mel115—1 μM unlabeled primers, 0.5 μM labeled primers; Mel113—2 μM unlabeled primer, 1 μM labeled primer. Mix 3 Mel114 and Mel110—4 μM unlabeled primers, 2 μM labeled primers; Mel103—0.5 μM unlabeled primer, 0.25 μM labeled primer. PCRs were undertaken using the Qiagen Multiplex PCR kit. 1 μl of extracted DNA (~50 μg/ml) was used as the template. PCR conditions for all three multiplex assays were as follows—96°C for 15 min, followed by 35 cycles of 96°C/30 s, 60°C/90 s, 72°C/90 s, followed by a final cycle of 72°C for 10 min.

Final PCR products were diluted 1:10 with double distilled water before electrophoresis on a Life Technologies ABI3130xl genetic analyzer (Life Technologies, Paisley, UK) using a GS500 Rox size standard. Allele calls were scored and manually checked using Life Technologies Gene‐mapper 4.0 software.

### 2.4 Genotyping quality control

We regenotyped a random selection of 5% of all extracted DNA samples tested. All microsatellite data were subjected to analysis by Microchecker v2.2.3 (van Oosterhout, Hutchinson, Wills, & Shipley, 2004) for the presence of genotyping errors and null alleles.

### 2.5 Population genetic clustering

We determined standard population genetic indices of diversity from the microsatellite data using GENEPOP v4.2 (Rousset, 2008), namely number of alleles (*N*_a_), observed (*H*_O_), and expected (*H*_E_) heterozygosity and the inbreeding/fixation coefficient (*F*_IS_). Deviations of allele frequencies from Hardy–Weinberg equilibrium (HWE) were also assessed by GENEPOP v4.2.

To elucidate the genetic population structure of badgers across Ireland, we analyzed microsatellite data of all 454 badger samples in an admixture model in STRUCTURE v2.3.4 (Pritchard, Stephens, & Donnelly, 2000) without location prior. With so little known about any potential subpopulation's history in Ireland, ancient or recent divergence of all subpopulations from a common ancestral population were both plausible scenarios, as was the possibility of different founding populations being translocated to Ireland by human agency, as has previously been inferred (Frantz et al., 2014; O'Meara et al., 2012).

All latter scenarios would have had consequences for heterogeneity in observed patterns of genetic relatedness and divergence among extant subpopulations; therefore, we ran STRUCTURE models accounting for both correlated and independent allele frequencies and assessed which produced the highest log likelihood for the inferred best fitting values of *K*. To infer the best fitting number of subpopulations (*K*), we used the Δ*K* method of Evanno, Regnaut, and Goudet (2005) over consecutive values from *K* = 1 to *K* = 10 with a burn‐in of 50,000 and a Markov chain length of 100,000, for 20 iterations per *K* value. Convergence of key statistics along the burn‐in chain was assessed as per the STRUCTURE manual. We then extracted and analyzed the data using STRUCTURE Harvester (Earl & vonHoldt, 2012). Data for each *K* value (*n* = 20) were processed by the program CLUMPP (Jakobsson & Rosenberg, 2007), with final illustrations produced using DISTRUCT (Rosenberg, 2004). We assigned individual badgers which exhibited 85% of their genetic heritage, or greater, to specific STRUCTURE defined subpopulations. Assignment thresholds for other animal species have been set at a variety of other values—50% for the American badger (Kierepka & Latch., 2016b), 70% for red deer and jaguars (Dellicour et al., 2011; Wultsch et al., 2016), and up to 90% for other mustelids (Cegelski, Waits, & Anderson, 2003) and reptiles (Gaillard et al., 2017). Given the reduced genetic diversity of the European badger in Ireland and the general philopatry of the species (Pope et al., 2006), and the continuous sampling structure we employed, we decided it would be best to use a threshold of 85%.

We quantified genetic differentiation between inferred STRUCTURE populations by calculating pairwise *F*_ST_ values using FSTAT 2.9.3.2 (Goudet, 1995). Statistical significance of pairwise values was tested by permutation with corrections for multiple comparisons. Genetic differentiation between pairs of populations was also quantified using Jost's *D* statistic (Jost, 2008) calculated by the mmod package (Winter, 2012) in the R environment v3.2.2 (R Development Core Team, 2008). All population data were mapped using ArcGIS ArcMAP 10 using latitude and longitude coordinates based on the Irish Grid (ESRI, 2011).

The STRUCTURE clustering algorithm works by maximizing linkage disequilibrium between markers and Hardy–Weinberg equilibrium among individuals in assigned populations (Pritchard et al., 2000; Wilkinson, Haley, Alderson, & Wiener, 2011). In continuously distributed species wherein there is clinal genetic differentiation, with isolation by distance, the algorithm can assign populations arbitrarily, so care must be taken in interpretation of data (Frantz et al., 2009; Pritchard et al., 2000). In line with this concern, we opted to make use of an additional multivariate clustering algorithm (Frantz et al., 2009) that did not make assumptions about linkage disequilibrium and Hardy–Weinberg equilibrium. Discriminant analysis of principal components (DAPC) is such a method, more suited to the investigation of population substructure in continuously distributed species that exhibit clinal genetic variation (Jombart, Devillard, & Balloux, 2010). We implemented the DAPC method in the adegenet package (Jombart, 2008) in the R environment v 3.2.2. We first used the *find.clusters* function to assign individual samples to proposed subpopulations, retaining all 70 principal components to infer a range of possible clusters. We then applied the DAPC analysis function to the upper and lower values of this range—in both cases retaining 30 principle components and all linear discriminants to produce scatterplots of both upper and lower values of *K*. As with STRUCTURE outputs, all population data were mapped using ArcGIS ArcMAP 10 using latitude and longitude coordinates based on the Irish Grid (ESRI, 2011).

### 2.6 Landscape genetics analyses

In order to examine the influence of geographic distance, landscape variables, and biotic interactions on badger gene flow, we combined Mantel tests, multiple regression on distance matrices (MRM), and redundancy analysis (RDA). Analyses were conducted with the subset of individuals for which precise coordinates of sampling were available (*n* = 433).

### 2.7 Mantel tests and Multiple regression on distance matrices (MRM)

As a first step, we estimated interindividual genetic distances (Smouse & Peakall, 1999) using the R package PopGenReport (Adamack & Gruber, 2014). Next, we used ArcGIS to generate resistance surfaces (rasters) representing the hypothesized resistance a particular environmental feature poses to badger gene flow (McRae, 2006). These surfaces were generated for land cover, elevation, earthworm availability, and geographic distance.

To generate land cover surfaces, we used the CORINE land cover data set (EEA, 1995) and generated two types of resistance surfaces: (a) based on broad land cover categories (CORINE Level 1), we assigned low resistance to *forest and seminatural areas*, intermediate resistance to *agricultural areas,* and high resistance to *artificial surfaces*; (b) within said categories (CORINE Level 2), we made further distinctions: within artificial surfaces, we lowered resistance for *artificial, nonagricultural vegetated areas*; within agricultural areas, we assigned low resistance to *pastures* and high resistance to *arable lands* and within forest and seminatural areas, we assigned highest resistance to *open spaces with no vegetation*.

Overall, taking into account general observations on badger ecology (Byrne et al., 2012; Hammond et al., 2001), we assumed that open areas (whether artificial or natural) posed higher resistance to gene flow than areas with vegetation cover. Resistance ratios for land cover surfaces were varied (1:10:100 vs. 1:100:1,000 vs. 1: 100: 10,000), thus generating six surfaces in total. Details on these surfaces and ratios are available in Supporting Information Data S2. We obtained a digital elevation model from CGIAR (http://srtm.csi.cgiar.org/), and two surfaces were generated, one maintaining raw elevation (masl) and one using a threshold of 200 m above sea level to assign low vs. high resistance (1:100) because badgers tend to avoid elevations beyond this threshold (Byrne et al., 2012).

To obtain a resistance surface related to earthworm availability, we used MaxEnt (Phillips, Anderson, & Schapire, 2006) to generate a raster of earthworm relative habitat suitability (EHS; Merow, Smith, & Silander, 2013). We obtained available records of the species (*n* = 30) from the Global Biodiversity Information Facility, GBIF (http://www.gbif.org/), and reviewed relevant literature on the ecology of *Lumbricus terrestris* and related taxa (Marchán et al., 2015; Rutgers et al., 2016) in order to select environmental variables to use as input in MaxEnt analyses. Coordinates for all *L. terrestris* records used in the MaxEnt analysis are shown in Supporting Information Table S2.

As a result, we selected five environmental variables, including three bioclimatic variables: temperature seasonality (BIO4), maximum temperature of the warmest month (BIO5), annual precipitation (BIO12; http://www.worldclim.org/bioclim), and two soil variables: pH and silt % (http://www.isric.org/data/isric-wise-global-soil-profile-data-ver-31).

**Figure 3:**
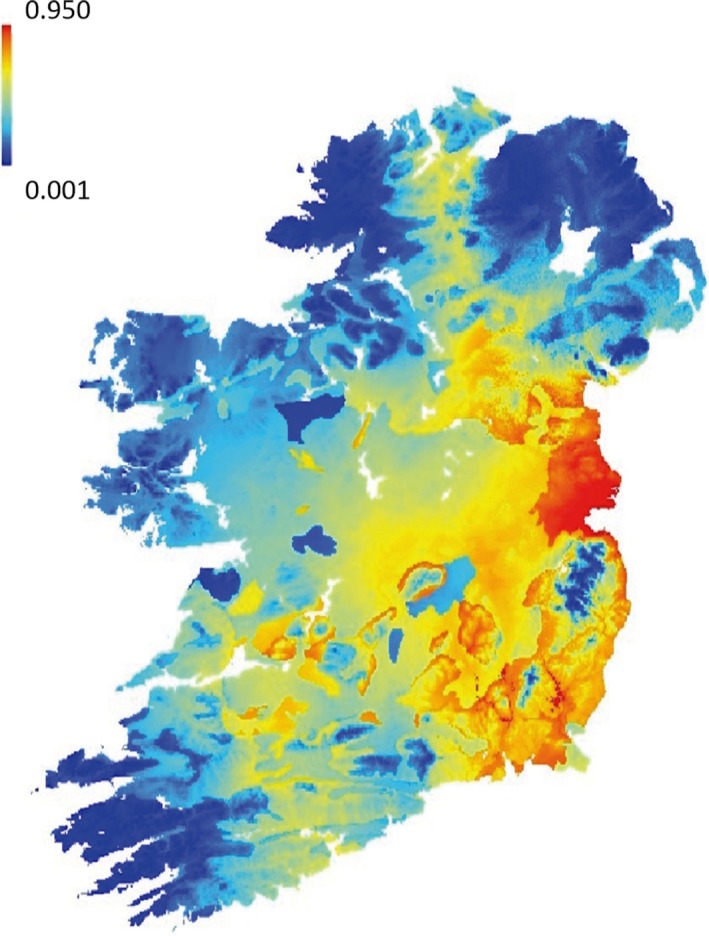
Habitat suitability map of Earthworm (*Lumbricus terrestris*) based on MaxEnt model. Map generated using MaxEnt logistic output; the values range from 0.001 to 0.950, where warmer colors indicate higher probability of presence and cooler colors indicate lower probability

Environmental variable rasters were tested for correlation using ArcGIS v 10.2 to avoid redundancy. Because no rasters were highly correlated (*r* < 0.80), they were used together as input in MaxEnt software, where analyses were run with default settings. Results of analyses showed the area under the curve (AUC) = 0.791 for the MaxEnt output model, which is considered an acceptable predictive accuracy (Araújo, Pearson, Thuiller, & Erhard, 2005). The variables with the highest percent contribution to the model were BIO 5 (31.3%) and BIO 12 (30.2%), followed by pH (20.2%), BIO 4 (9.8%), and silt (8.6%). Overall, there is an increase in habitat suitability with increasing values of BIO5 (max. temperature of warmest month), whilst the opposite was true for BIO12 (annual precipitation), where at values beyond ~770 mm, habitat suitability decreases, see Supporting Information Figure S2. The output habitat suitability raster is shown in Figure 3.

To test for isolation by distance (IBD), we generated a “flat” resistance surface in which all cells had the same value (=1). This is an alternative to using Euclidean distance that accounts for the finite size of the landscape (Dudaniec, Spear, Richardson, & Storfer, 2012).

From the generated resistance surfaces, we obtained resistance–distance matrices using the software Circuitscape 4.0 (McRae, Dickson, Keitt, & Shah, 2008). For land cover, elevation, and “flat” surfaces, we used default settings, which assume that these factors inhibit badger gene flow (i.e., raster values = resistance). For EHS, settings were modified so that raster values represented “conductance” (i.e., habitat suitability ranging from 0.001 to 0.950) because we expected high EHS to facilitate badger gene flow. Finally, to test whether the River Shannon acts as a barrier to gene flow, we used individual badger locations to generate a “barrier matrix” in which values indicated whether individuals had been sampled on the same side of the river (=1) or on opposite sides (=100). Once we obtained the resistance matrices, we used the package Ecodist (Goslee & Urban, 2007) in R, to conduct Mantel tests and MRM. Although the use of Mantel (and related) tests in landscape genetics has been criticized (Legendre & Fortín, 2010), recent simulations have shown that they are highly effective at detecting Isolation by distance (Kierepka & Latch, 2014). Hence, we performed a simple Mantel test between badger genetic distance and the “flat” matrix to test for IBD. Next, we performed partial Mantel tests on genetic and landscape resistance matrices, whilst controlling for “flat” distance. For both simple and partial Mantel tests, the significance of correlations (Spearman) was determined from 10,000 permutations. Partial Mantel tests identified the resistance matrices that showed significant correlation (*p* < 0.05) with genetic distance matrices. These were checked for correlation using the “cor” function in R, and only those without strong correlation (*r* < 0.8) were retained for MRM analyses. In MRM models, matrices were used as predictors of genetic distance. Model selection was done using a backward elimination approach, with a “threshold” *p* < 0.05. Significance of regression coefficients and R‐square values was assessed from 10,000 permutations.

### 2.8 Redundancy analysis

Ordination techniques, such as redundancy analysis (RDA), are increasingly used in landscape genetics studies given their power to detect effects of landscape resistance and barriers on gene flow (Kierepka & Latch, 2014). To apply RDA, we initially conducted a principal components analysis on genotypic data with the R package “adegenet” and retrieved the two‐first principal components to use as response variables. Predictors were point estimates of the landscape/biotic variables at the sampling site of individuals; to test for an effect of geography on gene flow, we used coordinates of sampling (latitude/longitude).

We conducted RDA in the R package vegan (Oksanen, Kindt, Legendre, & O'Hara, 2008). We first built a “full” model using all predictors together and checked for multicollinearity among them with the function “vif.cca.” Because all variance inflation factors were low (<5), we retained all predictors. Next, we used the function “ordistep” to conduct (backward) stepwise model selection and thus identify a “minimal” model. Having identified the minimal model, we conducted partial RDAs to estimate the amount of genetic variance explained solely by landscape/biotic variables controlling for geographic location (latitude/longitude) and that explained solely by geographic location controlling for landscape/biotic variables. All models were tested for significance using the function “anova.cca.”

## 3 RESULTS

### 3.1 Data quality assurance

Microsatellite retyping produced results identical to those initially obtained. Microchecker detected no evidence for genotyping errors or null alleles. Microsatellite allele calls for all samples are found in Supporting Information Data S1.

### 3.2 General population genetic indices

Indices of diversity, inbreeding fixation (*F*_is_) and tests for Hardy–Weinberg equilibrium (HWE) across all of Ireland, NI, RoI, and the five Irish populations identified by STRUCTURE (see below) are shown in Table 1. Across all of Ireland, deviations from HWE were observed across 12 of the 14 loci genotyped (Table 1A). Within the NI and RoI populations, seven and nine loci were out of HWE, respectively (Table 1B and C). Across the five subpopulations identified by STRUCTURE, between 1 and 2 loci deviated from HWE (Table 1D, E, F, G, and H). General population genetic indices of diversity across all of Ireland (see Table 1A) were similar to those described before by (O'Meara et al., 2012). More alleles per locus were observed in this study, 5.90 vs. 4.20, perhaps as a result of this study having surveyed with more microsatellite loci and across a wider geographic area, however, observed mean heterozygosity was similar—0.48 vs. 0.50. Observed diversity across NI and RoI was very similar (see Table 1B and C) with numbers of alleles per locus being 5.10 and 5.40, respectively, and observed mean heterozygosity 0.49 and 0.48, respectively.

**Table 1:** Population genetic summary statistics for all individual microsatellite loci and average across all loci for badgers from A All of Ireland, B Northern Ireland, C Republic of Ireland, D structure subpopulation IR1, E structure subpopulation IR2, F structure subpopulation IR3, G structure subpopulation IR4, H Structure Subpopulation IR5

		Locus														Mean
		Mel104	Mel106	Mel109	Mel111	Mel112	Mel117	Mel102	Mel105	Mel113	Mel115	Mel129	Mel103	Mel110	Mel114	
A	N	454	454	453	454	449	454	453	453	453	454	454	454	453	454	453.2
	Na	8	6	7	4	5	6	5	8	6	10	6	3	5	4	5.9
	He	0.68	0.52	0.37	0.29	0.47	0.73	0.52	0.68	0.69	0.54	0.67	0.45	0.58	0.66	0.56
	Ho	0.56	0.45	0.31	0.24	0.41	0.63	0.45	0.57	0.58	0.50	0.61	0.38	0.50	0.58	0.48
	Fis	0.18	0.14	0.18	0.16	0.14	0.14	0.12	0.16	0.17	0.07	0.09	0.16	0.14	0.12	0.14
	HW	a	a	a	a	a	a	n.s.	a	a	a	n.s.	a	a	a	
B	N	176	176	175	176	173	176	175	176	176	176	176	176	176	176	175.6
	Na	7	6	5	3	5	5	4	7	5	7	6	3	5	4	5.1
	He	0.70	0.55	0.33	0.28	0.47	0.73	0.49	0.70	0.64	0.53	0.65	0.44	0.63	0.66	0.56
	Ho	0.66	0.48	0.30	0.23	0.39	0.63	0.44	0.64	0.51	0.52	0.60	0.36	0.56	0.58	0.49
	Fi	0.05	0.13	0.09	0.19	0.17	0.14	0.10	0.09	0.20	0.01	0.08	0.18	0.12	0.12	0.12
	HW	a	n.s.	a	a	a	n.s	n.s.	n.s.	a	n.s.	n.s.	a	a	n.s.	
C	N	278	278	278	278	276	278	278	277	277	278	278	278	278	278	277.7
	Na	8	5	5	3	5	6	4	7	6	10	5	3	4	4	5.4
	He	0.64	0.49	0.39	0.30	0.48	0.72	0.52	0.60	0.68	0.55	0.67	0.46	0.49	0.66	0.55
	Ho	0.50	0.42	0.31	0.25	0.42	0.63	0.46	0.53	0.62	0.49	0.62	0.39	0.46	0.58	0.48
	Fis	0.22	0.15	0.22	0.15	0.13	0.12	0.11	0.12	0.09	0.11	0.08	0.15	0.06	0.11	0.13
	HW	a	a	a	a	a	n.s	n.s.	a	n.s.	a	n.s.	a	n.s.	a	
D	N	27	27	27	27	27	27	27	27	27	27	27	27	27	27	27
	Na	6	4	3	2	5	5	4	6	5	4	5	3	5	4	4.4
	He	0.78	0.59	0.17	0.50	0.76	0.70	0.30	0.66	0.61	0.55	0.43	0.52	0.77	0.62	0.57
	Ho	0.89	0.56	0.19	0.48	0.70	0.56	0.26	0.63	0.59	0.52	0.44	0.48	0.74	0.63	0.55
	Fis	−0.12	0.05	−0.07	0.03	0.07	0.21	0.13	0.04	0.03	0.06	−0.03	0.08	0.03	−0.01	0.04
	HW	n.s.	a	n.s.	n.s.	n.s.	a	n.s.	n.s.	n.s.	n.s.	n.s.	n.s.	n.s.	n.s.	
E	N	21	21	21	21	19	21	21	21	21	21	21	21	21	21	20.9
	Na	5	3	3	1	2	5	2	3	3	4	4	2	3	3	3.1
	He	0.56	0.57	0.52	0.00	0.23	0.67	0.31	0.64	0.29	0.40	0.58	0.50	0.29	0.65	0.44
	Ho	0.43	0.47	0.57	0.00	0.26	0.63	0.29	0.52	0.29	0.38	0.57	0.57	0.24	0.52	0.41
	Fis	0.24	0.16	−0.09	N/A	−0.13	0.15	0.10	0.19	0.03	0.04	0.01	−0.14	0.19	0.19	0.07
	HW	a	n.s.	n.s.	N/A	n.s.	n.s.	n.s.	n.s.	n.s.	n.s.	n.s.	n.s.	n.s.	n.s.	
F	N	11	11	11	11	11	11	11	11	11	11	11	11	11	11	11
	Na	3	2	2	2	3	4	2	3	3	3	3	2	3	3	2.7
	He	0.25	0.10	0.25	0.25	0.26	0.64	0.25	0.70	0.52	0.51	0.68	0.31	0.39	0.67	0.41
	Ho	0.27	0.10	0.27	0.27	0.27	0.55	0.27	0.72	0.55	0.45	0.64	0.36	0.36	0.64	0.40
	Fis	−0.07	0.00	−0.11	−0.11	−0.07	0.16	−0.11	−0.05	−0.06	0.11	0.07	−0.18	0.08	0.05	−0.02
	HW	n.s.	N/A	n.s.	n.s.	n.s.	n.s.	n.s.	n.s.	n.s.	n.s.	n.s.	n.s.	n.s.	n.s.	
G	N	14	14	14	14	14	14	14	14	14	14	14	14	14	14	14
	Na	6	2	3	2	4	6	3	3	5	6	4	3	4	3	3.9
	He	0.62	0.35	0.52	0.25	0.60	0.64	0.57	0.61	0.70	0.61	0.63	0.47	0.56	0.67	0.56
	Ho	0.57	0.29	0.64	0.29	0.43	0.71	0.57	0.43	0.71	0.71	0.64	0.43	0.64	0.71	0.56
	Fis	0.08	0.19	−0.25	−0.13	0.29	−0.11	0.00	0.31	−0.02	−0.17	−0.01	0.09	−0.15	−0.07	0.00
	HW	n.s.	n.s.	n.s.	n.s.	a	n.s.	n.s.	n.s.	n.s.	n.s.	n.s.	n.s.	n.s.	n.s.	
H	N	34	34	34	34	34	34	34	34	34	34	34	34	34	34	34
	Na	3	4	2	2	3	5	2	3	4	4	3	2	3	2	3
	He	0.37	0.56	0.50	0.44	0.55	0.73	0.50	0.42	0.58	0.65	0.64	0.41	0.26	0.43	0.50
	Ho	0.29	0.44	0.44	0.41	0.53	0.65	0.56	0.44	0.59	0.62	0.59	0.44	0.29	0.38	0.48
	Fis	0.20	0.22	0.13	0.07	0.03	0.11	−0.11	−0.04	0.00	0.06	0.10	−0.08	−0.12	0.12	0.05
	HW	a	n.s.	n.s.	n.s.	n.s.	n.s.	n.s.	n.s.	a	n.s.	n.s.	n.s.	n.s.	n.s.	

Notes

*F*_is_: Fixation index (Weir and Cockerham)—inbreeding of individuals relative to population; HW: significance of departure from Hardy–Weinberg Equilibrium at individual loci; *H*_e_: expected heterozygosity; *H*_o_: observed heterozygosity; *N*: number of individual badgers; *N*_a_: number of alleles observed at each locus.

a Significant difference (*p* < 0.05).

The fact that Northern Irish badgers were sampled after road traffic accident whilst their southern contemporaries were sampled after sett side trapping raises the possibility that behavioral differences potentially related to ranging may make direct comparison inappropriate. To address this, we compared allele frequency data between the Co. Down RTA population and another Co. Down population sampled sett side (Data not shown). *Z* tests corrected for multiple comparisons indicated there were no significant allele frequency differences.

### 3.3 Clustering and assignment methods

The independent allele frequencies STRUCTURE model outputs indicated a plateauing of log likelihood of *K* (L(*K*)) around *K* = 4 or 5 (Supporting Information Figure S3A). The Evanno Δ*K* plot showed two peaks, the highest at *K* = 2 and a lower one at *K* = 4 (Supporting Information Figure S3B). The correlated allele frequencies STRUCTURE model outputs indicated a plateauing of the log likelihood of *K* (L(*K*)) around *K* = 5 (Supporting Information Figure S4A). The Evanno Δ*K* plot showed two peaks, the largest at *K* = 2 and a smaller one at *K* = 5 (Supporting Information Figure S4B). The correlated allele frequency STRUCTURE model exhibited the highest mean log likelihood at all inferred values of *K* for 20 replicates compared to the independent allele frequencies model (Supporting Information Table S3). Consequently, we focused our efforts on the data from the correlated allele frequencies model. It has been noted before that the Evanno method can underestimate the true value of *K* when genetic differentiation is minimal between subpopulations, thereby preferentially finding the highest clustering hierarchy in a dataset (Waples & Gaggiotti, 2006).

**Figure 4:**
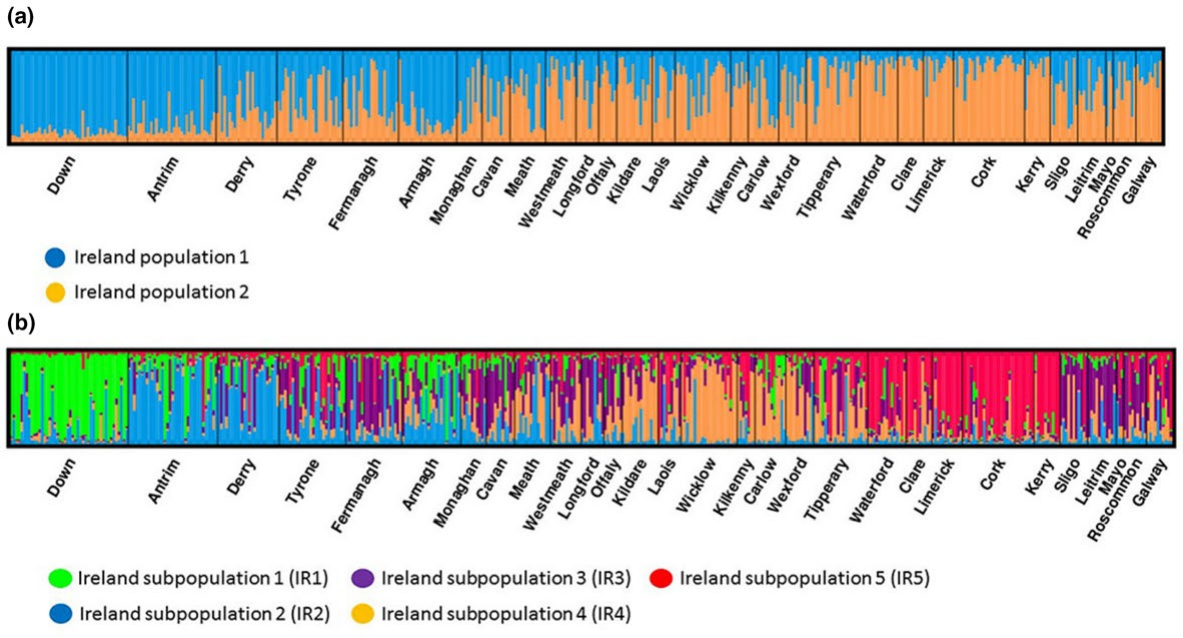
(a) STRUCTURE microsatellite admixture plot *K* = 2 for all Irish badgers. (b) STRUCTURE microsatellite admixture plot *K* = 5 for all Irish badgers

In another study involving the American badger, *Taxidea taxus,* two peaks have been observed using the Evanno method (Kierepka & Latch, 2016a), with the second being suggestive of further substructure (Evanno et al., 2005; Kierepka & Latch, 2016a). We chose therefore to investigate both *K* = 2 and *K* = 5 for the British and Irish data. DISTRUCT admixture plots of the *K* = 2 and *K* = 5 badger populations are shown in Figures 3a,b, respectively. At *K* = 2, Irish badgers separated into two distinct geographically distributed subpopulations (Figures 2a and 4a). In the northeast of the island, the Counties of Down, Antrim, and Armagh were home to most of this cluster (Figures 2a and 4a). The second subpopulation identified by the *K* = 2 analysis was distributed more widely, occupying the majority of the landmass of Ireland, being found primarily in western regions and the midlands. Admixed animals which could not be definitively assigned to either of the subpopulations were distributed across the midlands, and northwestern counties. A map of *K* = 2 population membership across Ireland indicating this distribution is presented in Figure 2. A distinct northeastern to southwestern cline in genetic differentiation appears to be a feature at this hierarchical level of clustering.

At *K* = 5, five Irish subpopulations (IR1‐IR5) were observed (Figure 4b). A map indicating spatial distribution of all five subpopulations is presented in Figure 2b. A total of 107 of the 454 animals genotyped were unambiguously allocated as belonging to one subpopulation on the basis of 85% of their genetic makeup having been assigned to a single subpopulation by the STRUCTURE admixture analysis. The remaining 347 genotyped animals did not meet these criteria and are represented as admixed animals in Figure 2b.

Subpopulation IR1 was primarily located in the northeastern County of Down, (Figures 2b and 4b). Subpopulation IR2 was largely localized to the northern Counties of Antrim and Derry (Figures 2b and 4b). Subpopulation IR3 was made up of few badgers and sporadically distributed Counties Monaghan, Fermanagh, Leitrim, Sligo, and Roscommon (Figures 2b and 4b). Subpopulation IR4 was distributed in the southeast, principally in Counties Wicklow, Kildare, and Wexford (Figures 2b and 4b). Subpopulation IR5 was primarily located in the southwestern Counties of Clare, Cork, Kerry, Limerick, and Waterford. Pairwise *F*_st_ and Jost's *D* statistics on subpopulation genetic differentiation for the *K* = 5 STRUCTURE analysis are shown in Table 2. All pairwise between subpopulation *F*_st_ calculations were observed to be significantly greater than zero. Admixed animals, which could not be definitively assigned to any of the five identified subpopulations, were distributed across the midlands and northwestern counties (Figures 2b and 4b).

**Table 2:** Pairwise *F*_st_ and Jost's between *K* = 5 Structure subpopulations—5 Irish subpopulations

	IR1	IR2	IR3	IR4	IR5
IR1	0	0.1489^a^/0.1850	0.1820^a^/0.2329	0.1597^a^/0.2480	0.1867^a^/0.2659
IR2	0.1489^a^/0.1850	0	0.2349^a^/0.2353	0.1927^a^/0.2362	0.2276^a^/0.2733
IR3	0.1820^a^/0.2329	0.2349^a^/0.2353	0	0.1243^a^/0.1375	0.1920^a^/0.2154
IR4	0.1597^a^/0.2480	0.1927^a^/0.2362	0.1243^a^/0.1375	0	0.1186^a^/0.1506
IR5	0.1867^a^/0.2659	0.2276^a^/0.2733	0.1920^a^/0.2154	0.1186^a^/0.1506	0

Note

For *F*_st_, significance was tested by 200 permutations in FSTAT.

a *p* < 0.05.

**Figure 5:**
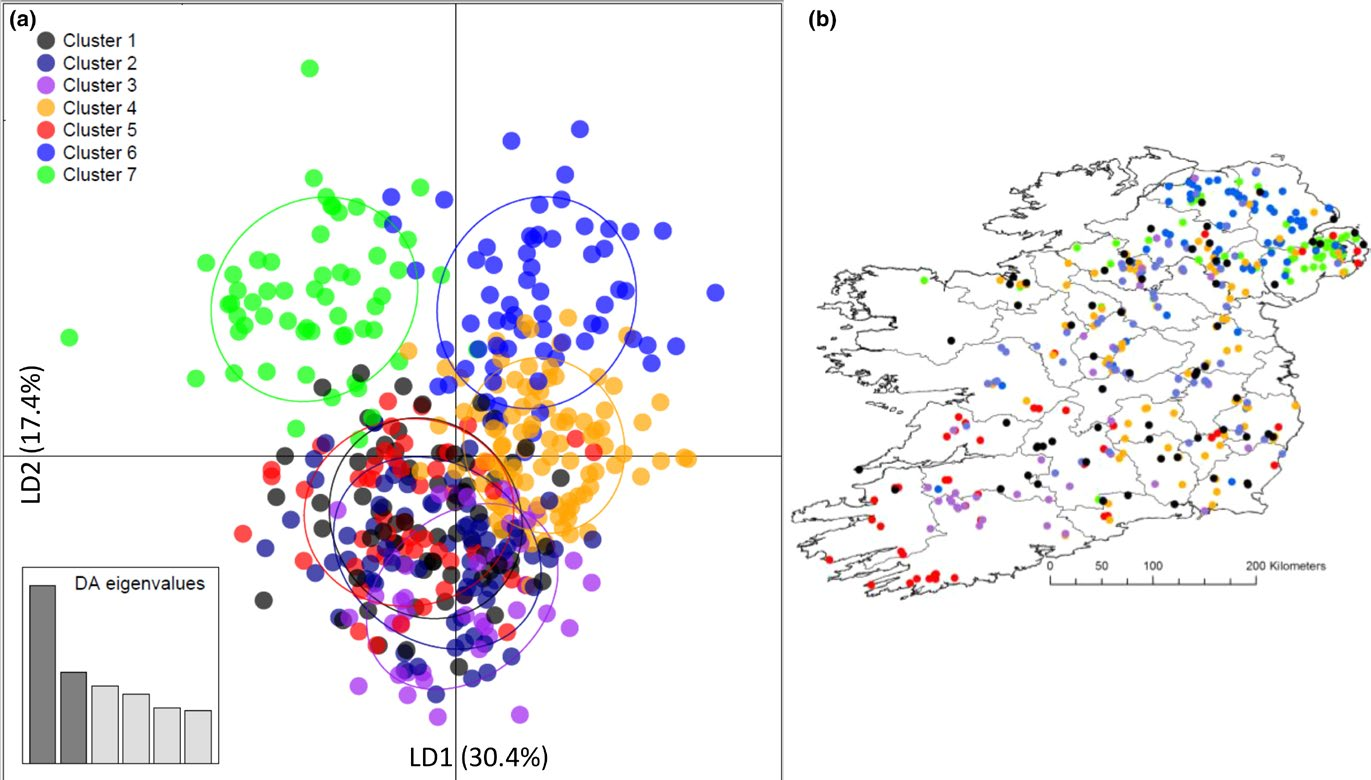
DAPC multivariate analysis of Irish badger genotype data. (a) DAPC *K* = 7 scatterplot of all individual badgers assigned to inferred seven subpopulation clusters; (b) DAPC *K* = 7 geo‐locations of all individual badgers and assigned subpopulation clusters

**Figure 6:**
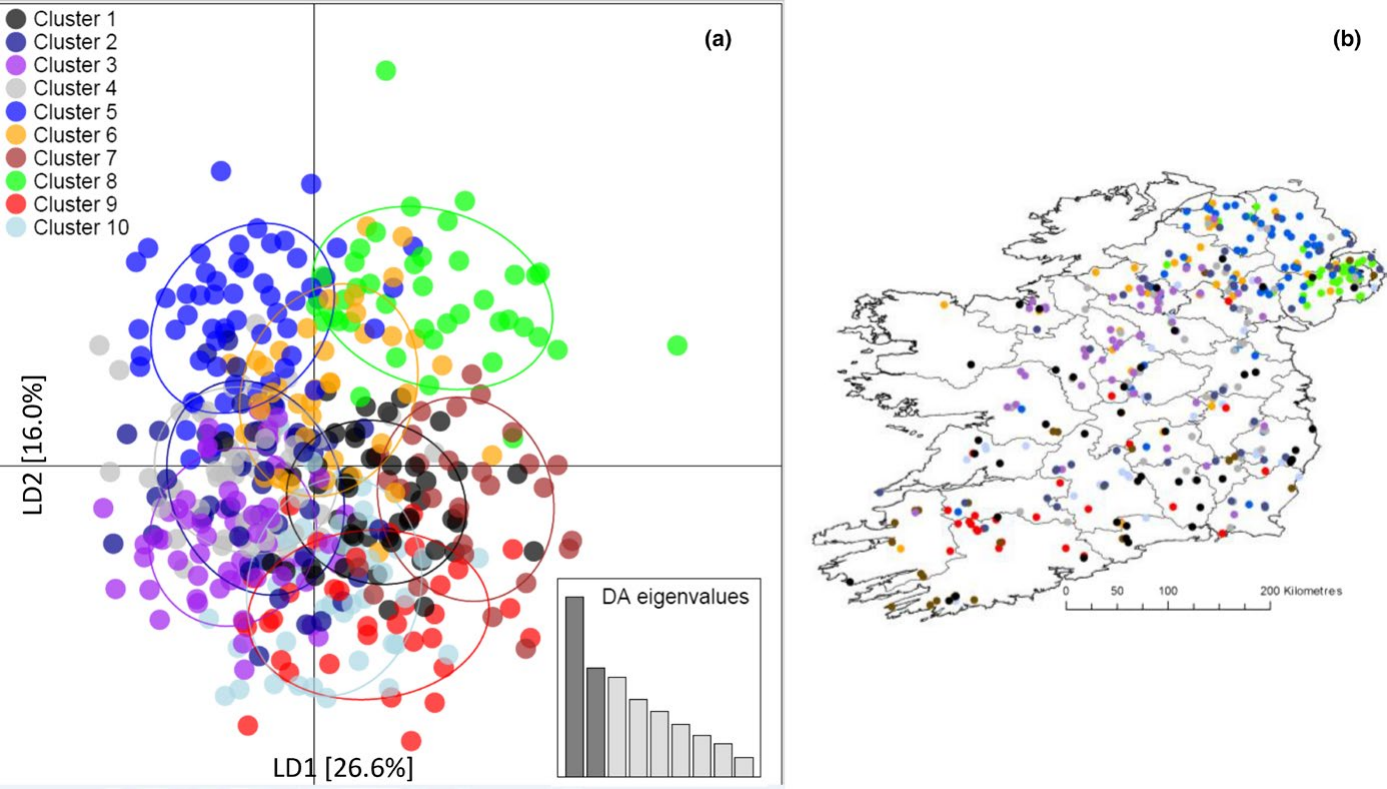
(a) DAPC *K* = 10 scatterplot of individual badgers assigned to inferred 10 subpopulation clusters; (b) DAPC *K* = 10 geo‐locations of all individual badgers and assigned subpopulation clusters

Supporting Information Figure S5 shows the curve of values of Bayesian information criterion (BIC) for each of the simulated values of *K* derived by find.clusters. The decreasing values of BIC begin to plateau at *K* = 7, reaching their lowest value at *K* = 10, before beginning to rise again (Supporting Information Figure S5). This type of pattern, with multiple possible values of *K*, is typical of “real world” scenarios involving continuously distributed species (Jombart, 2008). We therefore chose to apply the DAPC method to both the *K* = 7 and *K* = 10 assigned clusters. Scatterplots and associated geo‐locations for all subpopulations under both the *K* = 7 and *K* = 10 scenarios are illustrated in Figures 5 and 6, respectively. At *K* = 7, linear discriminant axis 1 (LD1) accounted for 30.4% of the observed genetic variance, whilst linear discriminant axis 2 (LD2) accounted for 17.4% of observed genetic variance (Figure 5a). At *K* = 10, LD1 accounted for 26.6% of the observed genetic variance, whilst (LD2) accounted for 16.0% of observed genetic variance (Figure 6a).

At *K* = 7, the DAPC scatterplot and geo‐location plot (Figure 5a,b) suggested that badgers from counties in the Republic of Ireland (Clusters 1–5) were more genetically homogeneous, exhibiting considerable overlap in both genetic and physical space. Conversely, Clusters 6 and 7 were primarily located in Northern Ireland and formed more distinct clusters in genetic space (Figure 5a,b). At *K* = 10, a similar pattern was observed with badgers from the Republic of Ireland exhibiting less genetic differentiation from each other than when compared to those from Northern Ireland (Figure 6a,b). However, in comparison with the *K* = 7 scenario, the *K* = 10 clusters exhibited a pattern more in keeping with a gradual cline in genetic variance both in genetic and physical space across the island.

### 3.4 Landscape genetic analyses

The simple Mantel test showed a significant correlation between genetic distance and the flat resistance surface (*r* = 0.24; *p* < 0.05), confirming IBD. When controlling for flat resistance–distance in partial Mantel tests, genetic distance showed significant positive correlation with raw elevation (*r* = 0.09; *p* < 0.05) suggesting that elevation inhibits gene flow.

No significant correlation was found with any land cover surface, the barrier matrix (River Shannon), or EHS. The results of the partial Mantel tests and correlations for all landscape factors are presented in Supporting Information Table S4. The final MRM model included only elevation and (flat) geographic distance and explained 7% of the variation in genetic distance (backward elimination was not performed as both predictors had *p*‐values <0.05; Table 3).

**Table 3:** Multiple regression on distance matrices (MRM)

Model	Variable	*β*	*p*‐Value
GD ~ GEO + ELV*	Geographic distance	1.631e−01	1e−04
	Elevation	1.278e−01	6e−04
*R* ^2^ = 0.070			1e−04

The model represents badger interindividual genetic distance as a function of geographic distance and elevation‐based resistance.

ELV: elevation; GD: Genetic distance; GEO: geographic distance.

After model selection in RDA, the minimal model included the variables: elevation, EHS, geographic location (latitude/longitude) and explained 40% of genetic variance (*F* = 72.235, *p* = 0.001). When controlling for geographic location with partial RDA, EHS and elevation only explained 2% of variance (*F* = 7.851, *p* = 0.001). In contrast, when controlling for EHS and elevation, geographic location explained 27% of variance (*F* = 97.135, *p* = 0.001).

## 4 DISCUSSION

In this study, we sought to better understand the population structure of the Irish badger and to determine how abiotic and biotic features of the Irish landscape affected gene flow and contributed to the extant population structure. Standard population genetic indices revealed island‐wide evidence of population substructure. The number of loci observed to be out of Hardy–Weinberg Equilibrium, and the higher values of the fixation index *F*_is_ across the whole island as a single unit, and when split into its two political units, indicated a lack of panmixia over large distances (Table 1A, B, and C). Indeed these data are consistent with a Wahlund effect and there being some subpopulation differentiation with limited connectivity. Similar findings have been noted before in badgers across Europe (Pope et al., 2006). Interestingly, RoI and NI exhibited very similar levels of heterozygosity and allelic diversity (Table 1A and B). RoI occupies approximately five times the landmass of NI, and whilst there are regional differences in land type and suitability for badgers (Byrne, Acevedo, Green, & O'Keeffe, 2014; Reid, Etherington, Wilson, Montgomery, & McDonald, 2012), one may have expected to see a more diverse RoI badger population. That this is not the case may be a result of the ongoing culling efforts in RoI (Sheridan, 2011). However, without baseline precull data, it is difficult to be certain this is the case. Wide scale culling has not been a feature of TB control schemes in Northern Ireland where badger populations have remained stable over many years (Reid et al., 2012).

Regarding the inferred population differentiation, STRUCTURE analysis indicated two levels of hierarchical clustering in the microsatellite data. At *K* = 2, there was an apparent northeastern to southwestern cline in badger genetic differentiation. From the data we present in this study, the reason for such structuring is not apparent. It may however have something to do with the way in which Ireland was populated by badgers in the past. The DAPC data for both *K* = 7 and *K* = 10 scenarios, particularly as pertaining to Northern Irish badgers being more genetically distinct than their southern contemporaries, support the STRUCTURE *K* = 2 inference that there is potentially something of interest about badger populations in Northern Ireland. Human‐aided transport of badgers from various distinct geographic locales into Ireland has been implied in wider European phylo‐geographic studies (Frantz et al., 2014; O'Meara et al., 2012). Such introductions may have left a lasting genetic signal within Irish badgers that still results in geographic structure.

At *K* = 5, we detected the presence of five Irish badger subpopulations occupying distinct geographic regions in Ireland. All five subpopulations exhibited significant levels of pairwise genetic differentiation, with IR1 and IR2 in Northern Ireland exhibiting the most differentiation from other subpopulations (Table 2). Subpopulation IR3 is very small and sporadically distributed across a wide range of Irish counties. The latter may be a STRUCTURE artifact that has arisen from the effects of IBD as previously discussed and described by Frantz et al. (2009). Given these problems, discussed above, with inference of clusters by STRUCTURE in continuously distributed species, it is pertinent to contrast our findings with those from the DAPC analyses. In both the *K* = 7 and *K* = 10 DAPC scenarios, we found evidence of a more gradual, clinal genetic differentiation across the island, which was particularly pronounced in the best supported (lowest BIC) *K* = 10 scenario. Such clinal variation would be in keeping with a species experiencing IBD across its range and is likely a more appropriate model of badger genetic variance than the one inferred by the *K* = 5 hierarchical STRUCTURE model. Indeed, the large numbers of animals that were found to exhibit admixed genetic heritage and no defined subpopulation membership in both the STRUCTURE *K* = 2 and *K* = 5 analyses lend credence to the DAPC supported clinal nature of badger genetic variation in Ireland.

Landscape genetics analyses showed an effect of geographic location/distance on genetic differentiation (i.e., IBD). This could indicate that Irish badgers are philopatric. Nevertheless, whilst IBD is consistent with, on average, limited dispersal, mark–recapture studies of Irish badgers have documented rare long‐distance dispersal of up to 22.1 km (Byrne, Quinn, et al., 2014). Both MRM and RDA models showed that elevation is related to genetic variation in badgers, which could indicate that gene flow is hindered by upland habitat. This is in accordance with data on badger habitat selection, with setts less commonly found in upland vegetation types (Byrne, Acevedo, et al., 2014; Hammond et al., 2001; Reid et al., 2012).

In terms of other environmental variables, only EHS appeared to affect genetic variance in badgers, but its influence was only detected through RDA and the variance explained was rather low (up to 2% combined with elevation). This pattern indicates that earthworm availability has a low influence on badger dispersal/gene flow, consistent with previous suggestions that Irish badgers have less‐specialized diets than populations elsewhere (Cleary et al., 2009). No other landscape features seemed to affect badger gene flow in Ireland, including the River Shannon. Although the River Shannon is a sizeable waterway, depth varies along its course and there are numerous man‐made structures such as bridges and canals that could allow badgers to cross. Indeed, it has been previously shown that rivers do not always represent impermeable barriers to badger gene flow (Frantz et al., 2010) or dispersal (Sleeman et al., 2009).

For a species with strict habitat requirements and limited dispersal ability, strong associations among habitats and genetic differentiation are expected (Kierepka & Latch, 2016b). However, despite evidence for limited dispersal in Irish badgers (i.e., IBD), and indications that aspects of badger ecology are affected by land cover (Byrne et al., 2012; Hammond et al., 2001), the latter was not related to badger genetic variation in landscape genetic analyses. Previous research has suggested that badgers in Ireland are less ecologically specialized than badgers in other parts of their range. For instance, several studies within Ireland have recorded setts in the vicinity of seemingly “unsuitable” habitats such as roadways, graveyards, and railways (Byrne et al., 2012), indicating the species is tolerant of human disturbance and can make use of a number of land cover types.

Our overall findings thus highlight the importance of spatial replication in landscape genetics studies (Castillo et al., 2016), as populations across species range may differ in their ecological interactions and requirements. Furthermore, the effect of particular landscape features on gene flow can strongly depend on the degree of landscape heterogeneity (Bull et al., 2011), which for Ireland could be classified as low.

## 5 CONCLUDING REMARKS

Our data demonstrate that geographic distance and elevation are pre‐eminent drivers of continuous, clinal, genetic variation in contemporary Irish badgers. These data can guide management of the species in Ireland, particularly in the context of controlling bovine tuberculosis in cattle. Knowledge of genetic population structure allows us to formulate testable hypotheses about how this level of partitioning might affect spatial disease patterns in the pathogen (Biek & Real, 2010).

Additionally, whilst philopatry appears to be the norm in the Irish badger and may contribute to maintenance of local *M. bovis* clusters, a small proportion of dispersers have been observed to move larger distances (Byrne, Quinn, et al., 2014). Given that there appear to be no major physical barriers to gene flow for badgers in Ireland, infected animals dispersing over a wider scale may therefore be a risk for disease spread. Our data and findings represent a strong foundation and timely opportunity to address these pertinent issues in the future.

## CONFLICT OF INTEREST

The authors declare no competing interest.

## AUTHORS’ CONTRIBUTIONS

JG participated in the design of the study, carried out statistical analysis, and drafted the manuscript. AB participated in the design of the study and drafted the manuscript. JL, EP, and GK carried out the molecular laboratory work. EC, JO'K, UF, and DO'M collected field samples. DE helped with statistical analysis. CM carried out field sampling and mapping. RB and RS participated in the design of the study and drafted the manuscript. AA conceived the study, participated in its design, drafted the manuscript, and performed statistical analysis and molecular laboratory work. All authors gave final approval for publication.

## DATA ACCESSIBILITY

All badger genotypic data are available from the Dryad Digital Repository: https://doi.org/10.5061/dryad.3r85690 as Supporting Information Data S1.
