# Supplementary material for: The population and landscape genetics of the European badger (Meles meles) in Ireland
Source: Ecol Evol. 2018 Sep 12;8(20):10233–46. doi: 10.1002/ece3.4498 (PMC6206220; doi:10.1002/ece3.4498)
Supplement: Supplementary file 1 [file ECE3-8-10233-s001.docx]

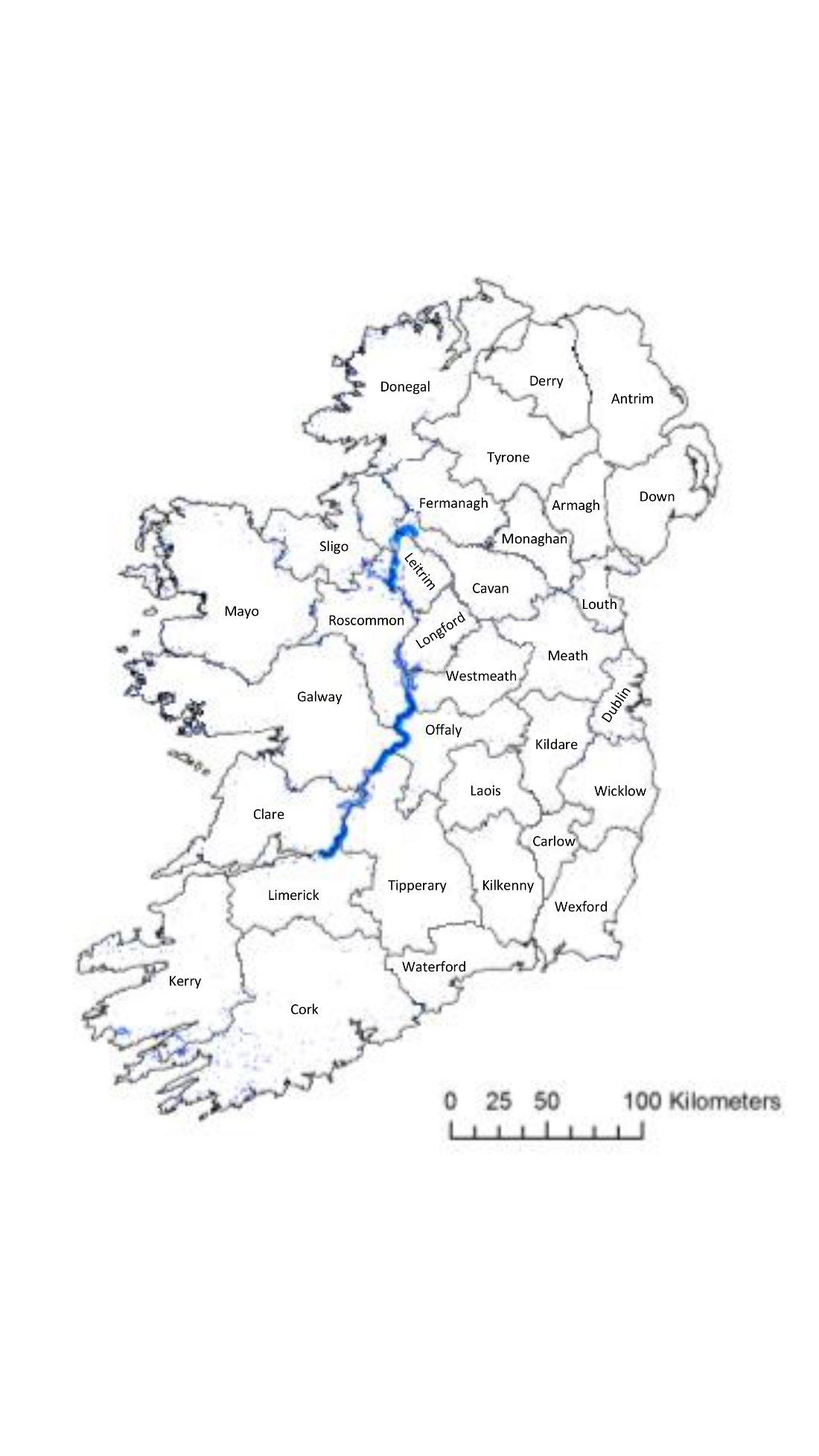


**Figure S1** – Map indicating geographic locations and names of all 32 Irish counties, and the position of the River Shannon highlighted in blue.


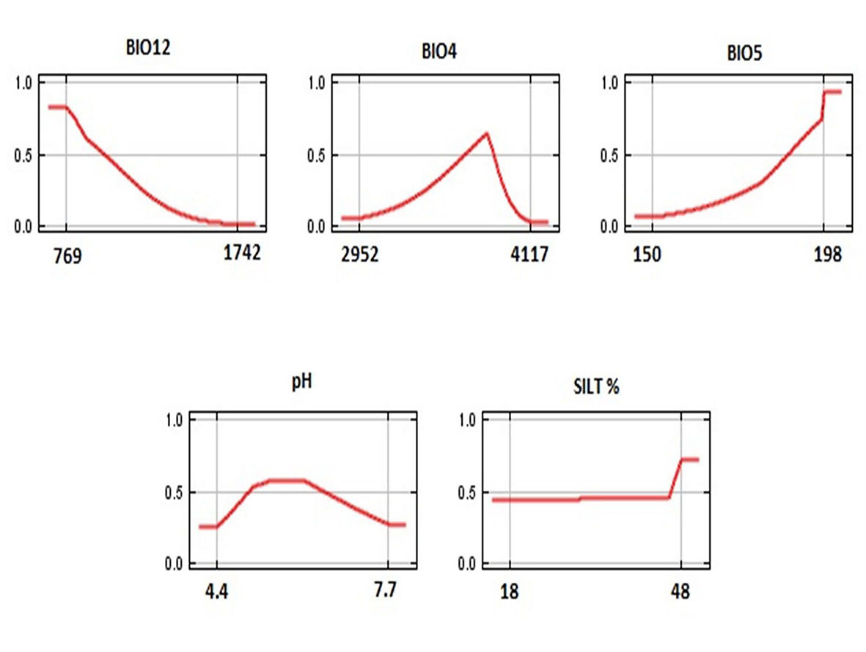


**Figure S2 -** Response curves of habitat suitability for five environmental variables based on MaxEnt model. The curves represent the dependence of habitat suitability on each environmental variable.

B

A


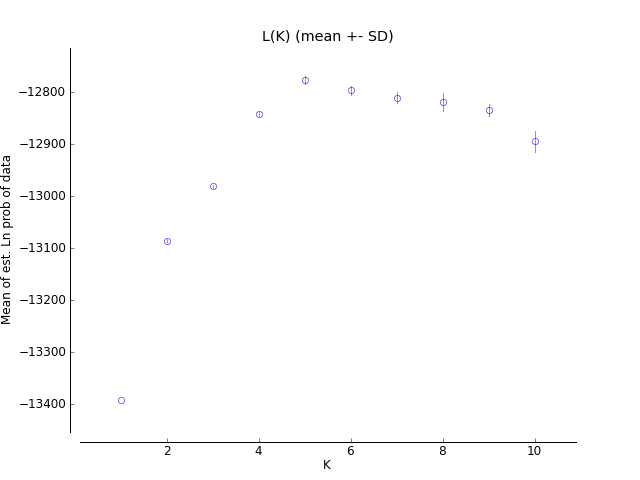

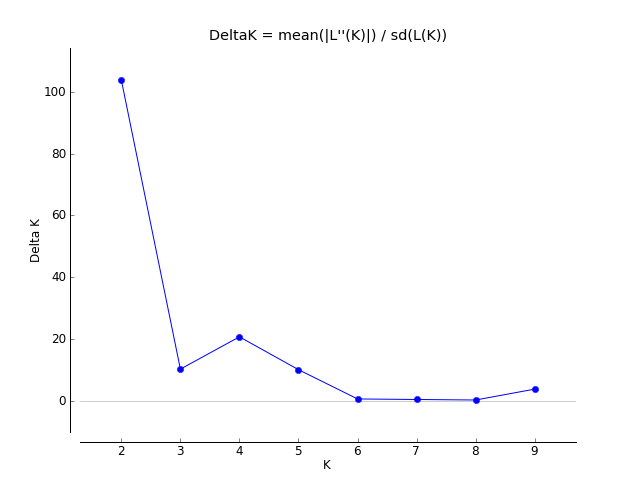


**Figure S3** **A**– STRUCTURE independent allele frequencies model output. Log likelihood of K plot from Structure Harvester (Earl and vonHoldt, 2012). **B -** STRUCTURE independent allele frequencies model output. Evanno ΔK method plot of rate of change of log probability between consecutive K values - from Structure Harvester (Earl and vonHoldt, 2012).

B

A


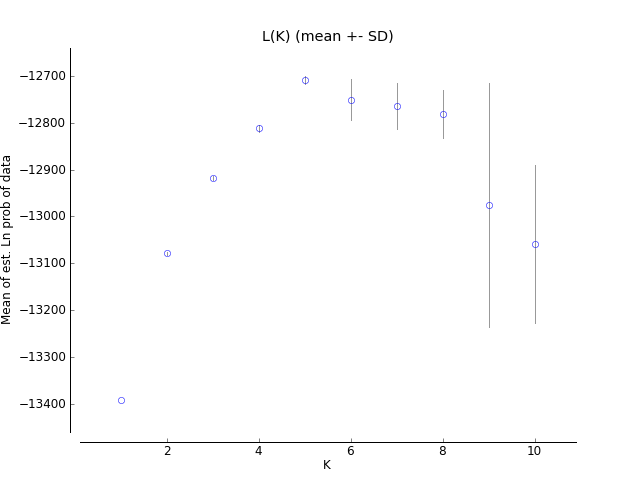

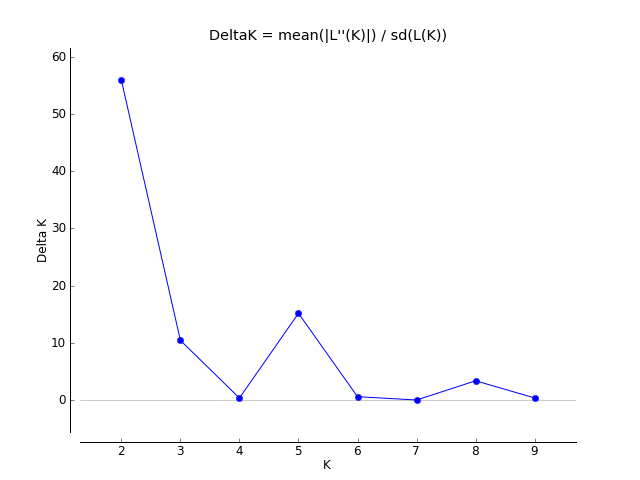


**Figure S4**  **A** – STRUCTURE correlated allele frequencies model output. Log likelihood of K plot from Structure Harvester (Earl and vonHoldt, 2012). **B -** STRUCTURE correlated allele frequencies model output. Evanno ΔK method plot of rate of change of log probability between consecutive K values - from Structure Harvester (Earl and vonHoldt, 2012).


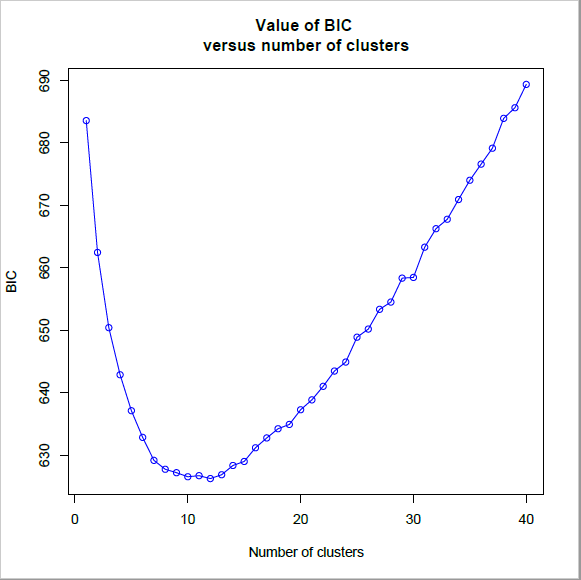


**Figure S5** – Adegenet function ‘find.clusters’ output after application to all Irish badger genotype data. Plot represents Bayesian Information Criterion for each simulated number of clusters (K).
