# Supplementary material for: The population and landscape genetics of the European badger (Meles meles) in Ireland
Source: Ecol Evol. 2018 Sep 12;8(20):10233–46. doi: 10.1002/ece3.4498 (PMC6206220; doi:10.1002/ece3.4498)
Supplement: Supplementary file 2 [file ECE3-8-10233-s002.docx]

| **County** | **No. of badgers sampled** |
| --- | --- |
| Antrim | 35 |
| Armagh | 23 |
| Carlow | 12 |
| Cavan | 11 |
| Clare | 10 |
| Cork | 28 |
| Derry | 24 |
| Down | 46 |
| Fermanagh | 22 |
| Galway | 10 |
| Kerry | 10 |
| Kildare | 14 |
| Kilkenny | 7 |
| Laois | 9 |
| Leitrim | 11 |
| Limerick | 12 |
| Longford | 9 |
| Mayo | 3 |
| Meath | 14 |
| Monaghan | 10 |
| Offaly | 7 |
| Roscommon | 9 |
| Sligo | 11 |
| Tipperary | 21 |
| Tyrone | 26 |
| Waterford | 15 |
| Westmeath | 12 |
| Wexford | 11 |
| Wicklow | 22 |

**Table S1** – Number of badger samples submitted per County across Ireland – 2011-2014.

| Latitude | Longitude | GBIF Identifier |
| --- | --- | --- |
| 51.79966 | -8.71101 | [doi.org/10.15468/dl.axpplj](http://doi.org/10.15468/dl.axpplj) |
| 52.29977 | -6.51067 | [doi.org/10.15468/dl.qqc8fg](http://doi.org/10.15468/dl.qqc8fg) |
| 52.42162 | -6.51614 | [doi.org/10.15468/dl.axpplj](http://doi.org/10.15468/dl.axpplj) |
| 52.44886 | -6.5027 | [doi.org/10.15468/dl.qqc8fg](http://doi.org/10.15468/dl.qqc8fg) |
| 52.46188 | -6.61261 | [doi.org/10.15468/dl.qqc8fg](http://doi.org/10.15468/dl.qqc8fg) |
| 52.47577 | -7.76522 | [doi.org/10.15468/dl.axpplj](http://doi.org/10.15468/dl.axpplj) |
| 52.56989 | -6.6269 | [doi.org/10.15468/dl.qqc8fg](http://doi.org/10.15468/dl.qqc8fg) |
| 52.7843 | -6.80023 | [doi.org/10.15468/dl.axpplj](http://doi.org/10.15468/dl.axpplj) |
| 52.82548 | -6.43651 | [doi.org/10.15468/dl.qqc8fg](http://doi.org/10.15468/dl.qqc8fg) |
| 52.87897 | -6.27723 | [doi.org/10.15468/dl.qqc8fg](http://doi.org/10.15468/dl.qqc8fg) |
| 52.92225 | -7.16814 | [doi.org/10.15468/dl.axpplj](http://doi.org/10.15468/dl.axpplj) |
| 53.01207 | -7.16523 | [doi.org/10.15468/dl.axpplj](http://doi.org/10.15468/dl.axpplj) |
| 53.05992 | -8.13498 | [doi.org/10.15468/dl.axpplj](http://doi.org/10.15468/dl.axpplj) |
| 53.09801 | -6.71687 | [doi.org/10.15468/dl.axpplj](http://doi.org/10.15468/dl.axpplj) |
| 53.31643 | -6.53252 | [doi.org/10.15468/dl.qqc8fg](http://doi.org/10.15468/dl.qqc8fg) |
| 53.31702 | -6.5316 | [doi.org/10.15468/dl.qqc8fg](http://doi.org/10.15468/dl.qqc8fg) |
| 53.31856 | -6.52945 | [doi.org/10.15468/dl.qqc8fg](http://doi.org/10.15468/dl.qqc8fg) |
| 53.32906 | -7.68632 | [doi.org/10.15468/dl.axpplj](http://doi.org/10.15468/dl.axpplj) |
| 53.49951 | -6.47873 | [doi.org/10.15468/dl.axpplj](http://doi.org/10.15468/dl.axpplj) |
| 53.5069 | -8.73931 | [doi.org/10.15468/dl.axpplj](http://doi.org/10.15468/dl.axpplj) |
| 53.58518 | -6.17397 | [doi.org/10.15468/dl.axpplj](http://doi.org/10.15468/dl.axpplj) |
| 53.68555 | -8.89371 | [doi.org/10.15468/dl.axpplj](http://doi.org/10.15468/dl.axpplj) |
| 53.81948 | -6.99893 | [doi.org/10.15468/dl.axpplj](http://doi.org/10.15468/dl.axpplj) |
| 53.90462 | -6.54013 | [doi.org/10.15468/dl.axpplj](http://doi.org/10.15468/dl.axpplj) |
| 53.94857 | -6.46236 | [doi.org/10.15468/dl.axpplj](http://doi.org/10.15468/dl.axpplj) |
| 53.95598 | -8.74696 | [doi.org/10.15468/dl.axpplj](http://doi.org/10.15468/dl.axpplj) |
| 54.04303 | -6.90929 | [doi.org/10.15468/dl.axpplj](http://doi.org/10.15468/dl.axpplj) |
| 54.09252 | -7.60345 | [doi.org/10.15468/dl.axpplj](http://doi.org/10.15468/dl.axpplj) |
| 54.94571 | -7.67032 | [doi.org/10.15468/dl.axpplj](http://doi.org/10.15468/dl.axpplj) |
| 55.17116 | -7.90669 | [doi.org/10.15468/dl.axpplj](http://doi.org/10.15468/dl.axpplj) |

**Table S2 –** Coordinates of *Lumbricus terrestris* records used in MaxEnt analysis.

| **K=2 Correlated Allele Freq. Model** | **K=2 Independent Allele Freq. Model** | **K=5 Correlated Allele Freq. Model** | **K=4 Independent Allele Freq. Model** |
| --- | --- | --- | --- |
| -12926.02 | -12940.42 | -12340.73 | -12543.11 |

**Table S3 –** Comparison of mean log likelihood for correlated vs uncorrelated allele frequency models run in STRUCTURE – Mean log likelihood values derived from n=20 replicate runs per K value for K=2 and K=5 for the correlated allele frequency model, K=2 and K=4 for the uncorrelated allele frequency model.

| Resistance surface | Description | r (p-value) |
| --- | --- | --- |
| ELVRAW | Raw elevation (masl) | **0.098 (0.000)** |
| ELV200 | Elevation 200m thresold | 0.035 (0.22) |
| LCOV1C | Land cover CORINE level 1 (see TSx) | 0.007 (0.781) |
| LCOV1CA | Land cover CORINE level 1 (see TSx) | 0.008 (0.770) |
| LCOV1CB | Land cover CORINE level 1 (see TSx) | 0.012 (0.650) |
| LCOV2C | Land cover CORINE level 2 (see TSx) | 0.001 (0.968) |
| LCOV2CA | Land cover CORINE level 2 (see TSx) | 0.003 (0.906) |
| LCOV2CB | Land cover CORINE level 2 (see TSx) | 0.000 (0.973) |
| SHANN | River Shannon Barrier | -0.005 (0.854) |
| EHS | Earthworm Habitat Suitability | 0.051 (0.073) |

**Table S4 -** Results of Partial Mantel tests among resistance surfaces and badger genetic distance. Table shows correlation coefficient (r) and p-value when partialling out geographic distance. Bold=significant correlation
